# Predictors of Efficacy of Janus Kinase Inhibitors in Patients Affected by Ulcerative Colitis

**DOI:** 10.3390/jcm13030766

**Published:** 2024-01-29

**Authors:** Giuseppe Cuccia, Giuseppe Privitera, Federica Di Vincenzo, Lucia Monastero, Laura Parisio, Luigi Carbone, Franco Scaldaferri, Daniela Pugliese

**Affiliations:** 1 Dipartimento di Medicina e Chirurgia Traslazionale, Università Cattolica del Sacro Cuore, L. Go A. Gemelli 8, 00168 Rome, Italy; giuseppe.cuccia@unicatt.it (G.C.); federica.divincenzo@unicatt.it (F.D.V.); lucia.monastero01@icatt.it (L.M.); franco.scaldaferri@policlinicogemelli.it (F.S.); 2 Dipartimento di Scienze della Salute, Università degli Studi di Milano, 20122 Milan, Italy; gpp.privitera@icloud.com; 3 IBD UNIT-CEMAD (Centro Malattie Apparato Digerente), Medicina Interna e Gastroenterologia, Fondazione Policlinico A. Gemelli IRCCS, 00168 Rome, Italy; laura.parisio@guest.policlinicogemelli.it; 4 UOC Pronto Soccorso, Medicina d’Urgenza e Medicina Interna, Ospedale Isola Tiberina Gemelli Isola, 00186 Rome, Italy; luigi.carbone@fbf-isola.it; 5 UOS Gastroenterologia, Ospedale Isola Tiberina Gemelli Isola, 00186 Rome, Italy

**Keywords:** JAK inhibitors, predictors, ulcerative colitis, biomarkers

## Abstract

Personalised medicine and the identification of predictors of the efficacy of specific drugs represent the ultimate goal for the treatment of ulcerative colitis (UC) in order to break the current therapeutic ceiling. JAK inhibitors are a new class of advanced therapies, orally administered, showing a good profile of efficacy and safety in both randomised controlled trials (RCTs) and real-world studies. Unfortunately, to date, it is not possible to draw the ideal profile of a patient maximally benefiting from this class of drugs to guide clinicians’ therapeutic choices. Baseline clinical activities and inflammatory biomarkers, as well as their early variation after treatment initiation, emerged as the main predictors of efficacy from post hoc analyses of RCTs with tofacitinib. Similar findings were also observed in the real-life studies including mainly patients with a history of pluri-refractoriness to biological therapies. At last, a few new biomarkers have been explored, even though they have not been validated in large cohorts. This paper provides a review of the current knowledge on clinical variables and biomarkers predicting response to JAK inhibitors in UC.

## 1. Introduction

Inflammatory bowel disease (IBD), that is, ulcerative colitis (UC) and Crohn’s disease (CD), is a systemic, chronic, relapsing–remitting disease. The pathophysiological mechanisms of IBD involve both genetic and environmental factors: the interplay between genetic susceptibility and environmental exposure is responsible for intestinal microbiota perturbations, gut epithelial barrier leaks and inappropriate activation of inflammatory pathways, causing microscopic and macroscopic bowel damage [1]. Although the exact pathophysiology of UC is yet to be fully elucidated, the current working model consists of a disproportionate immune response—primarily, but not solely, depending on T cell activity—characterised by the overproduction of inflammatory cytokines (including interleukin [IL]-13, IL-17, IL-23 and tumour necrosis factor [TNF]-α, amongst several others) that perpetrates inflammation and sustains the mechanisms that produce intestinal damage [2,3].

UC can affect the colon with different extensions (from isolated proctitis to pancolitis) and is clinically characterised by bloody diarrhoea, urgency and tenesmus, which can severely impair patients’ quality of life [4]. Biotechnological monoclonal antibodies—the first targeted therapies licensed for the treatment of steroid-dependent or -refractory IBD—act by blocking a single immunologic target, such as specific cytokines (TNF-α or IL-23) or gut-tropic integrins (e.g., α4β7). More recently, a new class of oral small molecules, named Janus kinase (JAK) inhibitors, has been introduced for UC treatment, including three drugs (tofacitinib, upadacitinib and filgotinib) that, despite sharing the same mechanism of action, differentiate from each other by their selectivity on different JAK subtypes [5].

The JAK family consists of four intracellular tyrosine kinases (JAK1, JAK2 and TYK2, which are ubiquitous in the body, and JAK3, present mainly in hematopoietic cells) that are coupled with several receptors involved in a number of immunological functions [6,7]. The binding of a cytokine to its corresponding receptor induces the dimerisation and activation of two JAK proteins. Activated JAKs promote the phosphorylation and subsequent activation of factors belonging to the signal transducer and activator of the transcription (STAT) family, which then act as transcription factors [8]. Tofacitinib is a nonselective JAK inhibitor, even though it exerts the greatest effect against the JAK1 and JAK3 isoforms, thereby blocking the production of several cytokines, including IL-2, IL-4, IL-6, IL-7, IL-12, IL-15 and IL-21 (but, notably, not TNF-α) [9,10]. On the other hand, upadacitinib and filgotinib selectively block JAK1, thus avoiding the involvement of JAK2, which participates in haematopoiesis [11], and JAK3, which contributes to maintaining intestinal barrier functions [12]. Furthermore, STAT5, which is predominantly activated by JAK2, is associated with a protective role for the intestinal epithelium, exerting an indirect effect in regulating the intestinal barrier [13,14]. While the inhibition of JAKs is an established strategy for the treatment of several immune and nonimmune diseases, STAT inhibitors—which act by inhibiting phosphorylation and dimerisation or by inducing degradation of STAT proteins—are still under investigation, mostly in the cancer field. Preclinical and clinical research has shown promising results for the use of STAT inhibitors as antitumour therapy [15]; whether they might also be used for the treatment of immune-mediated disease represents an undoubtedly intriguing speculation that needs to be explored in future research.

Translational works on animals and humans support the crucial role of the JAK-STAT pathway in IBD. Based on KEGG pathway enrichment analysis of differentially regulated genes, it was recently shown—at the single-cell level—that colonic samples from patients with CD exhibit significant upregulation of the ‘JAK-STAT signalling pathway’ in epithelial, immune and stromal cells [16]. Given the virtually ubiquitous expression of JAKs, it would appear reasonable to assume that JAK inhibitors act simultaneously on different cell types to exert their immunomodulatory functions. Indeed, it has been reported that tofacitinib functions by inhibiting the homing and activation of T cells in the gut [17]; however, this likely represents just one of several mechanisms of action by which JAK inhibitors work. Furthermore, it has also been shown that chronic inflammation is characterised by a differential methylation pattern of genes related to several metabolic pathways: of note, in a murine model of colitis, KEGG analysis showed enriched differential upstream methylation of genes related to the ‘JAK-STAT signalling pathway’ [18]. It would be interesting to systematically investigate whether JAK inhibitors also act by altering such methylation pathways.

Even with multiple therapeutic options available, patients with UC still have an estimated 10-year risk of colectomy up to 10% [19] and a risk of UC-related hospitalisation of almost 20% in the first 5 years after the diagnosis [20]. Long-term disease control is still challenging and an unmet need for more effective therapies or better strategies remains. Biological drugs, in particular anti-TNF-α drugs, are limited by a primary failure rate reaching up to 40% [21] and a rate of secondary failure of 15–20% per patient year [22,23,24,25]. Moreover, patients already exposed to anti-TNF-α have a reduced likelihood of achieving remission with a second-line drug, even with a different mechanism of action [26,27]. 

To overcome these issues, a growing interest has emerged in the following areas:

(1) Comparative research, aimed at identifying the drugs with the highest effectiveness [28];
(2) Personalised medicine, that is, the identification of clinical characteristics and/or biomarkers that can predict, ex ante, a patient’s response to a specific treatment.

In regard to predictors of response, a significant amount of data has been published so far; nevertheless, most of these markers have been identified ex post, and they have not been validated in large, prospective cohorts [29]. 

This review focuses on JAK inhibitors and aims to provide an overview of all the predictors of response identified for this class of drugs.

## 2. JAK Inhibitors: Efficacy in Randomised Controlled Trials

The OCTAVE programs included two parallel induction studies (OCTAVE 1 and 2), where patients (about 50% previously exposed to anti-TNF-α drugs) were randomised to tofacitinib 10 mg bis in die (BID) or placebo, followed by a maintenance study (OCTAVE SUSTAIN) enrolling week 8 responders who were randomised to receive tofacitinib 10 mg BID or 5 mg BID or placebo [30]. Tofacitinib was superior to placebo in inducing week 8 clinical remission (18.5% vs. 8.2%, *p* = 0.007, and 16.6 vs. 3.6%, *p* < 0.001, in OCTAVE 1 and 2, respectively). At week 52, the clinical remission rate was higher in both active arms compared to placebo (40.6% and 34.3% for the 10 mg BID and the 5 mg BID, respectively, vs. 11.1%, *p* < 0.001 for both comparisons) [30].

A similar design (two parallel induction studies, U-ACHIEVE induction and U-ACCOMPLISH, and one maintenance phase, U-ACHIEVE maintenance) was used in the upadacitinib phase 3 program, enrolling in similar proportions bio-naïve and bio-failure patients [31]. In the induction studies, a 45 mg daily dose of upadacitinib was superior to placebo in inducing clinical remission at week 8 (26% vs. 5% and 33% vs. 4%, *p* < 0.001 for both). After induction, week 8 responders were rerandomised into three arms: upadacitinib 30 mg daily, 15 mg daily or placebo. At week 52, patients treated with upadacitinib had a higher rate of clinical remission compared to placebo, regardless of the dose (52% for 30 mg, 42% for 15 mg vs. 12%, *p* < 0.001 for both comparisons) [31].

Finally, the efficacy of filgotinib was evaluated in the phase 2b/3 SELECTION program, comprising two induction studies (study A including bio-naïve patients and study B including bio-exposed patients) and one maintenance phase for induction responders [32]. In both induction studies, patients were randomised into three arms to receive filgotinib 100 mg daily, 200 mg daily or placebo. At 10 weeks, in study A, clinical remission was recorded in 19.1% and 26.1% with filgotinib 100 mg and 200 mg, respectively, compared to 15.3% with placebo (*p* = 0.34 and *p* = 0.02, respectively); in study B, clinical remission was observed in 9.5% and 11.5% of patients vs. 4.2% (*p* = 0.06 and *p* = 0.01, respectively). At week 58, a greater proportion of patients treated with filgotinib 200 mg and 100 mg achieved clinical remission compared to those with placebo (for study A, 37.2% vs. 11.2%, *p* < 0.0001, and for study B, 23.8% vs. 13.5%, *p* = 0.04) [32].

Table 1 summarises efficacy data obtained from phase 3 RCTs.

## 3. JAK Inhibitors: Effectiveness in Real-World Studies

Few data have been published so far on the effectiveness of JAK inhibitors in clinical practice, and most of them concern tofacitinib. 

Taxonera et al. performed a meta-analysis including 17 studies with a total of 1162 patients with UC receiving tofacitinib [33]. Thirteen studies reported data on previous exposure to biologics, with 793 patients (88.4%) exposed to at least one anti-TNF-α drug and 564 (62.9%) exposed to vedolizumab. Indeed, among studies reporting data on clinical remission (755 patients included), 34.7% of patients (95% confidence interval (CI), 24.4–45.1; nine studies) achieved clinical remission at 8 weeks, 47% (95% CI, 40.3–53.6; eight studies) in the interval between week 12 and 16 and 38.3% (95% CI, 29.2–47.5; five studies) at 6 months. Mucosal healing rate was explored in seven studies and reported in 41.9% of patients (95% CI, 18.1–65.6; three studies) at week 8 and 35.1% of patients (95% CI, 11.5–58.7; four studies) between week 12 and 16. Although underrepresented (11.6% of all patients included), bio-naïve patients seemed to have an increased rate of clinical response at week 8 (hazard ratio (HR) 1.38; 95% CI, 1.03–1.84, *p* = 0.03) [33]. 

With regard to upadacitinib, the largest real-life cohort published so far comes from an American prospective study, including patients affected by both UC (*n* = 44) and CD (*n* = 40); all of them had a history of failure to anti-TNF-α agents, and 17 patients with UC had also been previously exposed to tofacitinib. At week 4, 19 of 25 (76.0%) patients with baseline clinically active UC achieved clinical response and 18 of 26 (69.2%) were in clinical remission. At week 8, 23 of 27 (85.2%) patients with baseline clinically active UC achieved clinical response and 22 of 27 (81.5%) were in clinical remission. Out of nine tofacitinib-experienced patients with baseline clinically active UC, seven (77.8%) were in clinical remission at week 8 [34]. In another retrospective study, reporting data on 75 patients with UC receiving upadacitinib (all of whom previously exposed to biologics or small molecules, with 30% already exposed to tofacitinib), 64.0% were in steroid-free clinical remission between weeks 8 and 16; among 26 patients with available endoscopic evaluations, after a median of 23.3 weeks, 61.5% and 34.6% achieved endoscopic response and remission, respectively. Notably, tofacitinib-experienced patients were reported to have a comparable rate of steroid-free clinical remission compared to tofacitinib-naïve patients (*p* = 0.30) [35].

Finally, in a Scottish retrospective study including 91 patients, 67% of whom were naïve to both biologics and small molecules, treatment with filgotinib (median duration of 39 weeks, interquartile range (IQR) 23–49) induced clinical remission in 71.9% (41/57) and 76.4% (42/55) of patients at weeks 12 and 24, respectively. In addition, normalisation of C-reactive protein (CRP) was achieved by 62 of 71 patients (87.3%) and 40 of 45 patients (88.9%) at week 12 and 24, respectively; faecal calprotectin ≤ 250 mg/g was recorded in 82.8% of patients (48/58) at week 12 and 79.5% (35/44) at week 24 [36].

## 4. Safety

Although the most common adverse events (AEs) related to JAK inhibitors are mild, serious and opportunistic infections, cardiovascular events and venous thromboembolism (VTE) have also been reported [30,37].

In a worldwide post-marketing surveillance study, including all the safety reports for tofacitinib in patients with UC, among 12,103 included AEs, the most frequently reported were infections (relative risk (RR): 3.28 per 100 patients years (PYs)), vascular disorders (RR: 1.26 per 100 PYs), respiratory disorders (RR: 0.74 per 100 PYs), neoplasms (RR: 0.55 per 100 PYs) and cardiac disorders (RR: 0.5 per 100 PYs) [38].

The most frequently reported infections in patients with UC were nasopharyngitis and herpes zoster. Of note, the estimated reporting rate for serious infections in patients with UC receiving tofacitinib (3.28 per 100 PYs) was observed to be higher than that reported in patients with rheumatoid arthritis (RA) [38]. In all phase 2 and 3 trials and open-label extension, patients treated with tofacitinib presented a higher, dose-dependent risk of developing herpes zoster (65 patients, 5.6% in the study population); however, they tended to be noncomplicated and in most cases did not result in permanent discontinuation of therapy [39]. Increasing age (10-year increments), prior anti-TNF exposure and lower body weight were identified as significant risk factors for herpes infections [40]. The incidence of serious herpes zoster in patients exposed to filgotinib was very low, irrespective of the dose [41]. With regard to upadacitinib, only three cases were reported during the CELEST trial [42], while data from long-term follow-up registries and real-world data are not yet available.

Sandborn et al., in a 4.4-year follow-up, reported an incidence rate (IR) of nonmelanoma skin cancer (NMSC) of 0.7 (95% CI, 0.3–1.2) in patients with UC receiving tofacitinib [43]. In a post hoc analysis of three randomised, placebo-controlled studies involving 1124 UC patients, prior NMSC (hazard ratio (HR) 9.09; *p* = 0.0001), anti-TNF-α failure (3.32; *p* = 0.0363) and age (HR per 10-year increase: 2.03; *p* = 0.0004) were identified as independent risk factors for NMSC [44]. In a recent meta-analysis, considering 82,366 person years of exposure to JAK inhibitors from clinical trials and long-term extensions across all indications, the malignancy IR was 1.25 per 100 person years; notably, no significant differences were observed when comparing JAK inhibitors to placebo and methotrexate, but JAK inhibitors were associated with a significantly higher risk of all malignancies compared to anti-TNF-α therapies (IR ratio (IRR) 1.50; 95% CI 1.16 to 1.94) [45].

In the OCTAVE program, the concentration of low-density lipoprotein (LDL), high-density lipoprotein (HDL) and total cholesterol increased after induction [30], whereas Sandborn et al. reported no clinically meaningful changes in the LDL/HDL ratio in 4.4 years of follow-up [43]. Clinical trials of tofacitinib in UC comprehensively showed an increased risk of major adverse cardiovascular events (MACE), with an incidence rate of 0.2 (95% CI, 0.1–0.6). A post hoc analysis from the ORAL Surveillance study reported a nearly significant higher MACE risk with tofacitinib 5 mg BID (8.3%; 17/204) and 10 mg BID (7.7%; 17/222) compared to TNF inhibitors in patients with rheumatoid arthritis and a history of atherosclerotic cardiovascular disease (HR 1.98, 95% CI 0.95–4.14, *p* = 0.196); the highest risk was associated with age > 50 years and at least one additional cardiovascular (CV) risk [46]. Conversely, Curtis et al., in a comparative observational study, reported an IR of 0.51 (95% CI, 0.31–0.79; 3903.3 PY) in UC patients receiving anti-TNF, compared to 0.28 (95% CI, 0.11–0.59; 2459.3 PY) in the tofacitinib UC clinical trial overall cohort, thus suggesting a safer CV profile for tofacitinib compared to anti-TNF-α drugs [47].

**Figure 1:**
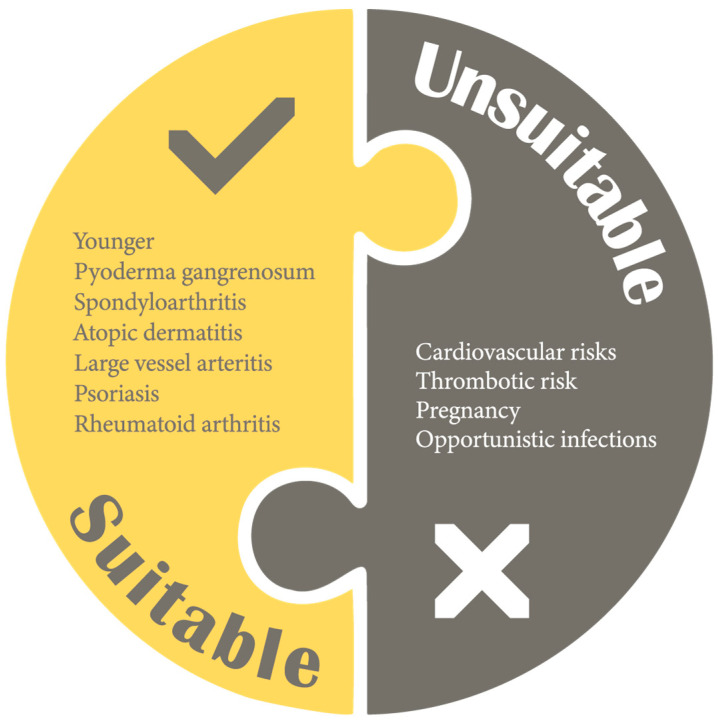
Factors to consider when prescribing a JAK inhibitor.

In February 2019, the Food and Drug Administration (FDA) placed a “black box warning” on the higher dose of tofacitinib (10 mg BID) in rheumatoid arthritis, owing to a potentially higher risk of VTE and an increased risk of all-cause mortality [48]. However, the increased risk was observed in patients > 50 years old with ≥1 cardiovascular risk factor or a previous history of malignancies [49]. In a post hoc analysis of the OCTAVE clinical trials (phase 2, 3 and open-label extension studies), the overall incidence rates of deep venous thrombosis and pulmonary embolism were 0.04 events/100 PYs (95% CI 0–0.23) and 0.16 events/100 PYs (95% CI 0.04–0.41), respectively [50]. Figure 1 summarizes factors to consider when prescribing a JAK inhibitor.

## 5. Predictors of Response to JAK Inhibitors in Ulcerative Colitis

### 5.1. Predictions Based on Baseline Characteristics

Post hoc analyses of the OCTAVE trials [30] have identified potential predictors of response to tofacitinib [51,52,53,54].

Lees et al. [51] aimed to create models capable of predicting the outcomes of tofacitinib-treated UC patients, analysing the data from the OCTAVE induction studies. They adopted both traditional statistics and machine learning techniques (specifically, random forest analyses) and identified the following variables with the highest predictive value for clinical response: baseline, week 2 and 4 partial Mayo score (PMS) and partial Mayo subscores (PMSb), baseline and week 4 CRP and total cholesterol serum levels. Baseline and week 2 variables predicted clinical response at week 4 with 84–87% accuracy and at week 8 with 74–79% accuracy; when including baseline, week 2 and week 4 variables, the accuracy in predicting clinical response at week 8 reached 85–87%; of note, logistic regression and random forest yielded comparable results, with a slight superiority shown by logistic regression. 

A more recent post hoc analysis of the OCTAVE induction studies [52] showed that CRP drop and PMS reduction at week 4 were associated with better outcomes in terms of clinical response (per unit odds ratio (OR) 0.86, 95% CI, 0.77–0.97, *p* = 0.0147, and OR 0.51, 95% CI 0.46–0.57, *p* < 0.0001, respectively), clinical remission (per unit OR 0.78, 95% CI, 0.67–0.91, *p* = 0.0020, and OR 0.46, 95% CI 0.40–0.54, *p* < 0.0001, respectively) and endoscopic improvement (per unit OR 0.66, 95% CI, 0.58–0.75, *p* < 0.0001, and OR 0.66, 95% CI 0.60–0.73, *p* < 0.0001, respectively) at week 8. CRP but not PMS was also associated with week-8 endoscopic remission (OR 0.62, 95% CI 0.53–0.73, *p* < 0.0001). With regard to week-8 clinical remission, receiver operating characteristic (ROC) curve analysis showed an area under the curve (AUC) of 0.72 for observed CRP at week 4 (proposed optimal threshold 0.94 mg/L; sensitivity 73.4%, specificity 62.4%), of 0.82 for observed PMS at week 2 (proposed optimal threshold 3; sensitivity 81.1%, specificity 70.8%) and of 0.85 for PMS at week 4 (proposed optimal threshold 2; sensitivity 84.4%, specificity 72.8%). Furthermore, a history of anti-TNF-α failure had a poor predictive value (AUC 0.51–0.7) for all efficacy outcomes. Among tofacitinib induction nonresponders who maintained treatment at the dose of 10 mg BID, a reduction in PMS at week 8 was associated with a clinical response at week 16 (univariate: per unit, OR, 0.59, 95% CI 0.46–0.75, *p* < 0.001).

Two other post hoc analyses [53,54] explored the presence of potential predictors of endoscopic remission and clinical remission by analysing data obtained from the OCTAVE Sustain study [30]. Sandborn et al. [53] showed that a baseline Mayo endoscopic subscore (MES) of 2 (vs. 3, OR 1.60, 95% CI, 1.06–2.44, *p* = 0.03), a post-induction PMS < 2 (vs. ≥2, OR 1.92, 95% CI, 1.27–2.90, *p* = 0.002) and older age (continuous; per 10 years; OR, 1.19; 95% CI, 1.02–1.39, *p* = 0.02) were associated with an increased probability of clinical remission at 52 weeks. In contrast, higher post-induction CRP values (per unit; OR, 0.94; 95% CI, 0.89–0.99, *p* = 0.03) and post-induction oral corticosteroid use (OR, 0.63; 95% CI, 0.42–0.96, *p* = 0.03) negatively impacted the likelihood of remission. Similar findings emerged when exploring the probability of loss of response during OCTAVE Sustain [53], with post-induction CRP values (per unit HR 1.02, 95% CI 1.01–1.04; *p* = 0.008) and post-induction corticosteroid use (HR 1.94, 95% CI 1.51–2.48; *p* < 0.0001) being associated with a worse outcome. The confidence interval is relatively narrow for CRP with an HR close to 1 because of the small number of patients with a high CRP value enrolled in the OCTAVE Sustain study. Conversely, baseline albumin (continuous; per unit HR 0.74; 95% CI, 0.55–1.00, *p* = 0.5), older age (continuous; per 10 years HR, 0.88; 95% CI, 0.80–0.96, *p* = 0.0048) and a post-induction PMS < 2 (vs. ≥2, HR 0.72; 95% CI, 0.56–0.93, *p* = 0.010) were protective against a loss of response. Concomitant extra-intestinal manifestations (HR 2.61, 95% CI 1.42–4.81, *p* = 0.0021) were associated with an increased risk of loss of response only for patients treated with 5 mg BID compared to 10 mg BID in the maintenance phase; it should be noted that only 22 patients with extra-intestinal manifestations were included in the maintenance study. 

The second post hoc analysis explored the capability of MES to predict clinical and endoscopic outcomes at 24 and 52 weeks [54]. A post-induction MES of 0 was associated with a numerically higher rate of clinical response at 52 weeks compared to an MES value of 1 (61.9% vs. 36.5% for tofacitinib 5 mg BID and 75.0% vs. 54.2% for tofacitinib 10 mg BID, respectively). An MES = 0, compared to MES =1, was associated with a significantly lower risk of both treatment failure (HR 0.29; 95% CI 0.10–0.84; *p* = 0.0231) and loss of response (HR 0.26; 95% CI 0.08–0.81; *p* = 0.0209). Other differences in outcomes were not statistically significant.

Moving to real-world studies, in a retrospective study from the U.K. enrolling patients with UC receiving tofacitinib, primary nonresponse assessed at week 8 was recorded in 26% of patients and independently associated with younger age at tofacitinib initiation (OR 1.04, 95% CI 1.01–1.07, *p* = 0.014) and a higher baseline CRP value (OR 0.292, 95% CI 0.12–10.655, *p* = 0.004); notably, bio-experienced status was not associated with a decreased likelihood of achieving clinical response or remission [55].

Data obtained from the ENEIDA registry, including 113 multi-refractory patients with UC receiving tofacitinib (69% of whom were previously exposed to more than three biologics), showed a reduced probability of clinical remission at week 8 in patients with higher PMS at week 4 (OR 0.2, 95% CI 0.1–0.4) [56]. Similar findings emerged for clinical remission at week 16 for patients who had higher PMS at weeks 4 (OR 0.5; 95% CI 0.3–0.7) and 8 (OR 0.2; 95% CI 0.1–0.5). Higher PMS at week 8 was also associated with a lower persistence on tofacitinib therapy (HR 1.5, 95% CI 1.3–1.6). 

In a Canadian multicentre, retrospective study enrolling 334 patients with UC receiving tofacitinib, among initial responders de-escalated to 5 mg BID, an increased likelihood of loss of response was recorded among those with a baseline MES of 3 (adjusted HR (aHR) 3.60, 95% CI 1.70–7.62, *p* = 0.001) and previous failure to biologic therapies (aHR 3.89, 95% CI 1.28–11.86, *p* = 0.02) [57]. Conversely, endoscopic remission at week 8, defined as MES = 0 (aHR 0.41, 95% CI 0.20–0.80, *p* = 0.009), but not just endoscopic improvement, defined as MES = 1 (aHR 1.26, 95% CI 0.61–2.60, *p* = 0.53), protected from a loss of response. These data suggest careful consideration for possible dose reduction at the end of the induction phase in patients with high baseline MES, especially if endoscopic remission is not achieved after 8 weeks of therapy [58].

### 5.2. Predictions Based on Faecal Calprotectin

Various studies have been conducted over the years to investigate the utility of faecal calprotectin in predicting patients’ outcomes on biologic therapy and to guide physicians’ decisions [59,60,61,62].

In their 2020 study, Dulai et al. tested the performance of faecal calprotectin to integrate rectal bleeding score (RBS) and stool frequency score (SFS) in order to predict endoscopic activity in patients with UC who had started a targeted therapy. Data on RBS, SFS and MES, obtained from the clinical trials of infliximab, golimumab, vedolizumab and tofacitinib in UC, were combined with the data from a systematic review of the operating properties of faecal calprotectin. In patients who achieved RBS = 0 and SFS = 0–1 after induction, a cut-off of faecal calprotectin of 50 ± 10 μg/g could correctly identify patients with endoscopic improvement (true negative 55.5%) and patients with moderate-to-severe endoscopic activity (true positive 20.5%), with a false negative rate of 4.5%. Conversely, in patients who still had RBS = 2–3 and SFS = 2–3 after induction, a cut-off of faecal calprotectin of 250 μg/g could correctly identify patients with endoscopic improvement (true negative 7.9%) and patients with moderate-to-severe endoscopic activity (true positive 68.4%), with a false positive rate of 2.1%. A similar performance for faecal calprotectin was also described in similar clinical scenarios during maintenance therapy [63].

Finally, from the U.K. multicentre experience already mentioned in the previous section, a significant drop in median faecal calprotectin level from baseline to week 8 was observed in UC patients responding to tofacitinib treatment (504 µg/g, IQR 289–1050, vs. 117 µg/g, IQR 35–432; *p* = 0.071), while no such difference was recorded in primary nonresponders (1074 μg/g, IQR 427–1912, vs. 1099 μg/g, IQR 540–1643, *p* = 0.130) [55].

### 5.3. Predictions Based on New Biomarkers

A few studies have investigated novel biomarkers for predicting response to JAK inhibitors.

A longitudinal study identified a potential predictive role for a panel of 53 aberrant methylation profiles correlated to tofacitinib treatment [64]. The study included 31 young Caucasian patients with moderate-to-severe UC with a baseline MES of 2 or 3 (22 patients had been exposed to anti-TNF alpha, while 15 had already been on therapy with ustekinumab). Patients underwent a blood draw at baseline (T1) and a subsequent blood draw at 8 weeks of therapy (T2). In addition, an endoscopic examination was performed at T2 to distinguish responders from nonresponders. Fifty-three methylated CpG loci associated with clinical, biochemical and endoscopic response could be traced: gene expression analysis showed lower expression of hypermethylated FGFR2 (fibroblast growth factor receptor 2) and LRPAP1 (a chaperone protein capable of binding calmodulin) and higher expression of hypomethylated OR2L13 (olfactory receptor) at T1 in responder patients. Of note, the methylations were shown to have good stability over time, up to 2 years [64].

Preliminary data from a prospective study conducted on 16 bio-naïve patients with moderate-to-severe UC with MES ≥ 2 receiving tofacitinib revealed a cluster of 65 genes expressed in the colonic mucosa that correlate with response to tofacitinib [65]; of note, the hub gene showed higher accuracy (*p* < 0.001) and independence from changes in inflammatory biomarkers of disease. In addition, the identified biomarker was significantly reduced at week 8 compared to baseline (*p* = 0.0004) in responder patients but not in nonresponders. 

In the context of ‘-omics’ techniques, which are proving more and more helpful in predicting response to biological therapies, a U.S. study using proteomic analysis in patients with psoriasis and on therapy with tofacitinib or etanercept was able to develop, via machine learning, an algorithm for predicting therapeutic response at 12 weeks [66]. In the study, blood samples from 266 patients enrolled for a phase 3 trial of tofacitinib (*n* = 138) vs. etanercept (*n* = 128) in psoriatic disease were analysed [67]. Patients underwent an initial sampling at baseline and a second sampling at week 4, showing a protein pattern capable of predicting response (defined as Psoriasis Area Severity Index of 75) to tofacitinib at 12 weeks (area under the receiver operating characteristic curve (AUROC) = 78%). Although these data do not directly pertain to UC, it is still possible to imagine a future application of these techniques in the world of IBD.

Finally, the group of Sasson et al. conducted a cross-sectional gene sequencing and cell profiling study of 35 patients, including 10 with UC, 15 with immune checkpoint inhibitor (ICI) colitis and 10 on immune checkpoint inhibitor therapy but without colitis [68]. Among patients with ICI colitis, the case of a 61-year-old man is described who, after rapid resolution of symptoms by faecal transplantation, experienced disease relapse refractory to conventional therapy and was then treated with tofacitinib. A dominance of interferon-gamma-producing CD8+ tissue memory T cells in immune checkpoint inhibitor colitis was identified in the study: notably, these cells were significantly reduced following tofacitinib therapy. These results, although with the considerable limitations of the study, suggest, on the one hand, that the therapeutic range of tofacitinib expands to this type of disease, and, on the other hand, they allow us to formulate new hypotheses regarding the immunological patterns on which small molecules act and, therefore, to speculate about potential biomarkers to identify.

Table 2 summarises the findings regarding proposed predictors of response to tofacitinib in patients with UC. 

## 6. Conclusions

Personalised or precision medicine represents one of the most valuable strategies for improving the efficacy of therapies in every field of medicine. It is based on the possibility of predicting, ex ante, the response to a specific drug in every single patient with a given disease and tailoring the therapeutic approach accordingly. The best example is represented by oncology, where, in clinical practice, treatment decisions for several types of cancers are driven by tumour-specific genetic or epigenetic changes or specific biomarkers [71,72]. With regard to IBD, several efforts have been made so far, especially after the introduction of anti-TNF-α drugs, whose effectiveness is impacted by a significant risk of primary or secondary nonresponse [21,22]. Moreover, the advent of new classes of advanced therapies has also raised the question of which drug is to be preferred over the others, in particular for bio-exposed patients. Comparative research and in particular head-to-head trials are helpful in satisfying these unmet needs, but the identification of patient-specific biomarkers could also reduce trial costs (via early identification of patients at high risk of failure) and improve their external validity.

Over the last years, JAK inhibitors have progressively occupied a relevant position in the therapeutic armamentarium of UC due to their good profile of effectiveness, rapidity of action, brief half-life and oral administration. Effectiveness in other immune-mediated conditions and peculiar safety warnings for some special populations (e.g., elderly or pregnant women) are two clinical aspects that influence, one way or another, physicians’ choices. So far, only a few potential predictors of response have been identified for JAK inhibitors, most of which are represented by clinical variables, coming from post hoc analyses of trials and not externally validated; only a few biomarkers have been investigated, mostly in small cohorts without prospective validation. A general grasp of the data presented so far seems to show that, owing to the rapidity of actions characterizing JAK inhibitors, early changes in clinical and biological markers (e.g., patient-reported outcome, CRP, faecal calprotectin) appear to be reliable predictors of response; however, this will need to be confirmed with large, prospective studies. Moreover, too early assessments could induce the inappropriate withdrawal of a therapy, which might exert its effect more slowly in a specific subset of patients.

Similar considerations should be applied regarding the new biomarkers explored so far, including epigenetic and genetic changes and proteomic profiles; data show encouraging results, but the patient cohorts are small and do not always just include patients affected by UC; hence, further confirmatory studies are necessary. Moreover, the feasibility of these analyses in clinical practice could be impaired by costs, the time needed for results and the availability of specific techniques at each centre. 

In conclusion, early clinical and biochemical response to JAK inhibitors seems to represent a prognostic marker for the clinical course of UC patients. The identification of new biomarkers, their validation in prospective studies and their integration with clinical, biochemical and endoscopic features, potentially through artificial intelligence techniques, represent one of the most relevant challenges for the future in the IBD field.

## Figures and Tables

**Table 1:** Phase 3 efficacy trials of JAK inhibitors in ulcerative colitis.

Randomised Controlled Trial			Patients Enrolled	Dose (Patients Randomised)	Concomitant Corticosteroid	Previous Anti-TNF-α Exposure	Primary Outcome	Response (*p* Value vs. Placebo)
tofacitinib	Sandborn WJ et al. (2017) [30]	OCTAVE Induction 1	598	Placebo (122)	47.5%	53.3%	Remission ^1^ at 8 Weeks	8.2%
				10 mg (476)	45.0%	53.4%		18.5% (*p* = 0.007)
		OCTAVE Induction 2	541	Placebo (112)	49.1%	58.0%		3.6%
				10 mg (429)	46.2%	54.5%		16.6% (*p* < 0.001)
		OCTAVE Sustain	593	Placebo (198)	50.5%	46.5%	Remission ^1^ at 52 weeks	11.1%
				5 mg (198)	51.0%	45.5%		34.3% (*p* < 0.001)
				10 mg (197)	44.2%	51.3%		40.6% (*p* < 0.001)
filgotinib	Feagan BG et al. (2021) [32]	SELECTION Induction A	659	Placebo (137)	24.8%	0.0%	Remission ^2^ at 10 weeks	15.3%
				100 mg (277)	24.2%	0.7%		19.1% (*p* = 0.3379)
				200 mg (245)	22.0%	0.0%		26.1% (*p* = 0.0157)
		SELECTION Induction B	689	Placebo (142)	35.9%	97.9%		4.2%
				100 mg (285)	36.1%	99.3%		9.5% (*p* = 0.0645)
				200 mg (262)	35.9%	98.9%		11.5% (*p* = 0.0103)
		SELECTION Maintenance	664	Placebo (89)	39.3%	39.3%	Remission ^2^ at 58 weeks	13.5%
				100 mg (172)	43.6%	38.9%		23.8% (*p* = 0.420)
				Placebo (98)	40.8%	44.8%		11.2%
				200 mg (199)	39.1%	46.2%		37.2% (*p* < 0.001)
upadacitinib	Danese S et al. (2022) [31]	U-ACHIEVE Induction	473	Placebo (154)	39.6%	50.6%	Remission ^3^ at 8 weeks	4.5%
				45 mg (319)	38.9%	52.7%		26.0% (*p* < 0.001)
		U-ACCOMPLISH	515	Placebo (174)	41.4%	51.1%		4.0%
				45 mg (341)	35.2%	50.4%		33.4% (*p* < 0.001)
		U-ACHIEVE Maintenance	451	Placebo (149)	40.3%	54.4%	Remission at 52 weeks	12.1%
				15 mg (148)	37.2%	48.0%		42.6% (*p* < 0.001)
				30 mg (154)	37.0%	47.4%		51.9% (*p* < 0.001)

^1^ Total Mayo score ≤ 2 with no subscore > 1 and an RBS = 0; ^2^ MES ≤ 1, RBS = 0 and at least a 1-point decrease in SFS from induction baseline for a subscore of 0 or 1; ^3^ Adapted Mayo score ≤ 2 with SFS ≤ 1 and not greater than baseline, RBS = 0 and MES ≤ 1 without friability. Abbreviations: RBS, rectal bleeding score; MES, Mayo endoscopic subscore; SFS, stool frequency score.

**Table 2:** Studies predicting the efficacy of Tofacitinib.

First Author (Year)	Study Design (Patients Enrolled)	Predictors/Markers Investigated	Timing of Measurement and Comparison of Investigated Predictors/Markers	Main Predicted Outcome	Effectiveness Measure
Lees CW et al. (2021) [51]	Post hoc analysis of OCTAVE Induction 1 and 2 studies (905) [30]	PMS, PMSb ^1^, CRP and total cholesterol serum levels	Multivariate and machine learning models including baseline vs. week 2 vs. week 4 variables	Clinical response ^2^ at week 8	Accuracy of 85–87%
Dubinsky MC et al. (2023) [52]	Post hoc analysis of OCTAVE Induction 1 and 2 studies (905) [30]	CRP and PMS	Baseline vs. week 4	Clinical response ^2^ at week 8	CRP: OR = 0.86; 95% CI, 0.77–0.97, *p* = 0.0147
					PMS: OR = 0.51; 95% CI, 0.46–0.57, *p* < 0.0001
				Clinical remission ^3^ at week 8	CRP: OR = 0.78; 95% CI, 0.67–0.91, *p* = 0.0020
					PMS: OR = 0.46; 95% CI, 0.40–0.54, *p* < 0.0001
				Endoscopic improvement ^4^ at week 8	CRP: OR = 0.66; 95% CI, 0.58–0.75, *p* < 0.0001
					PMS: OR = 0.66; 95% CI, 0.60–0.73, *p* < 0.0001
				Endoscopic remission ^5^ at week 8	CRP: NS
					PMS: OR = 0.62; 95% CI, 0.53–0.73, *p* < 0.0001
Sandborn WJ et al. (2022) [53]	Post hoc analysis of OCTAVE Sustain (487) [30]	PMS, MES, age, CRP and steroid use	Post-induction PMS < 2 vs. ≥2, baseline MES 2 vs. 3, baseline age, post-induction CRP, post-induction steroid use	Remission ^3^ at week 52	Post-induction PMS: OR 1.92; 95% CI, 1.27–2.90, *p* = 0.002
					Baseline MES: OR 1.60, 95% CI, 1.06–2.44, *p* = 0.03
					Older age, per 10 years: OR, 1.19; 95% CI, 1.02–1.39, *p* = 0.02
					Higher CRP, per unit: OR, 0.94; 95% CI, 0.89–0.99, *p* = 0.03
					Steroid use: OR, 0.63; 95% CI, 0.42–0.96, *p* = 0.03
Lee SD et al. (2023) [54]	Post hoc analysis of OCTAVE Sustain (487) [30]	MES	Post-induction MES 0 vs. 1	Treatment failure at week 52	HR = 0.29; 95% CI 0.10–0.84; *p* = 0.0231
				Loss of response	HR = 0.26; 95% CI 0.08–0.81; *p* = 0.0209
Honap S et al. (2020) [55]	Retrospective (134)	Age and CRP	Baseline	Primary nonresponse at week 8	Younger age: OR = 1.04, 95% CI 1.01–1.07, *p* = 0.014
					Higher CRP: OR = 0.292, 95% CI 0.12–10.655, *p* = 0.004
Chaparro M et al. (2021) [56]	Retrospective (113)	PMS and RBS	Baseline vs. week 4 and vs. week 8	Clinical remission ^6^ at week 8	Higher PMS at week 4: OR 0.2, 95% CI 0.1–0.4
				Clinical remission ^7^ at week 16	Higher PMS at week 4: OR 0.5; 95% CI 0.3–0.7Higher PMS at week 8: OR 0.2; 95% CI 0.1–0.5
				Persistence on tofacitinib	Higher PMS at week 8: HR 1.5, 95% CI 1.3–1.6
Ma C et al. (2023) [57]	Retrospective (334)	MES and bio-exposed status	Baseline MES = 3 vs. <3, bio-naïve vs. bio-exposed status, endoscopic remission at week 8	Loss of response ^8^ at week 52 in patients de-escalated to 5 mg BID	MES: aHR = 3.60, 95% CI 1.70–7.62 *p* = 0.001
					Biologic exposure: aHR 3.89, 95% CI 1.28–11.86, *p* = 0.02
					Endoscopic remission: aHR = 0.41, 95% CI 0.20–0.80, *p* = 0.009
Joustra V et al. (2023) [64]	Prospective (31)	Genome-wide peripheral blood DNA methylation signatures	53 CpG methylated CpG loci at baseline	Tofacitinib response ^9^ at week 8	AUROC 0.74
Verstockt BV et al. (2020) [65]	Prospective (52)	Gene expression of biopsy sampling from the colonic mucosa	One cluster comprising 65 genes at baseline	Endoscopic response ^10^ at week 8–14	Accuracy of 100%, *p* < 0.001

^1^ Stool frequency, rectal bleeding and physician global assessment; ^2^ clinical response: 2- or 3-point decrease in PMS; ^3^ remission: total Mayo score ≤ 2 with no individual subscore > 1 and a rectal bleeding subscore of 0; ^4^ endoscopic improvement: MES = 0–1; ^5^ endoscopic remission: MES = 0; ^6^ treatment failure: increase from OCTAVE Sustain baseline total Mayo score of ≥3 points, plus an increase in rectal bleeding subscore of ≥1 point and an increase in MES of ≥1 point yielding an absolute MES of ≥2 after a minimum of 8 weeks in the study; ^7^ clinical remission: PMS < 2; ^8^ loss of response: increase in PMS ≥ 2 points with accompanying biomarker or endoscopic evidence of inflammation; ^9^ tofacitinib response: endoscopic response (decrease in MES ≥ 1 or UCEIS ≥ 2 compared to baseline) combined with clinical response (decreased total Mayo score ≥ 3 and ≥30% and decreased RBS ≥ 1 or absolute subscore ≤ 1, or 50% drop in SSCAI score) and/or biochemical response (≥50% decrease in CRP and FC or CRP ≤ 5.0 mg/L and FC ≤ 250 ug/g); ^10^ MES ≤ 1. Abbreviations: PMS, partial Mayo score; PMSb, partial Mayo subscores; CRP, C reactive protein; OR, odds ratio; CI, confidence interval; NS, not selected; MES, Mayo endoscopic subscore; HR, hazard ratio; RBS, rectal bleeding subscore; aHR, adjusted hazard ratio; FC, faecal calprotectin; AUROC, area under the receiver operating characteristic curve; UCEIS, Ulcerative Colitis Endoscopic Index of Severity [69]; SSCAI, Simple Clinical Colitis Activity Index score [70].

## Data Availability

Not applicable.
